# Age-related changes in synaptic markers and monocyte subsets link the cognitive decline of APP_Swe_/PS1 mice

**DOI:** 10.3389/fncel.2012.00051

**Published:** 2012-11-01

**Authors:** Gaëlle Naert, Serge Rivest

**Affiliations:** Laboratory of Endocrinology and Genomics, CHUQ Research Center and Department of Molecular Medicine, Faculty of Medicine, Laval UniversityQuébec City, QC, Canada

**Keywords:** Alzheimer's disease, bone marrow-derived microglia, monocytes, CCR2, BDNF, memory impairments

## Abstract

Alzheimer's disease (AD) is characterized by a progressive memory decline and numerous pathological abnormalities, including amyloid β (Aβ) accumulation in the brain and synaptic dysfunction. Here we wanted to study whether these brain changes were associated with alteration in the population of monocyte subsets since accumulating evidence supports the concept that the innate immune system plays a role in the etiology of this disease. We then determined the immune profile together with expression of genes encoding synaptic proteins and neurotrophins in APP_Swe_/PS1 mice and their age-matched wild-type (WT) littermates. We found that the progressive cognitive decline and the dramatic decrease in the expression of numerous synaptic markers and neurotrophins correlated with a major defect in the subset of circulating inflammatory monocytes. Indeed the number of CX_3_CR1^low^Ly6-C^high^CCR2^+^Gr1^+^ monocytes remained essentially similar between 5 weeks and 6 months of age in APP_Swe_/PS1 mice, while these cells significantly increased in 6-month-old WT littermates. Of great interest is that the onset of cognitive decline was closely associated with the accumulation of soluble Aβ, disruption of synaptic activity, alteration in the BDNF system, and a defective production in the subset of CX_3_CR1^low^Ly6-C^high^CCR2^+^Gr1^+^ monocytes. However, these memory impairments can be prevented or restored by boosting the monocytic production, using a short treatment of macrophage colony-stimulating factor (M-CSF). In conclusion, low CCR2^+^ monocyte production by the hematopoietic system may be a direct biomarker of the cognitive decline in a context of AD.

## Introduction

Alzheimer's disease (AD) is a progressive neurodegenerative disorder affecting the elderly. AD begins as mild short-term memory deficits and culminates in total loss cognition and executive functions. The two core pathological hallmarks are amyloid deposits and neurofibrillary tangles (Selkoe, 2002). These senile plaques appeared primarily in the cerebral cortex and hippocampus, leaving the brainstem and cerebellum essentially unaffected. Amyloid precursor protein (APP) is cleaved to produce beta-amyloid (Aβ) peptides, namely Aβ_1–40_ and Aβ_1–42_. Aβ oligomerizes to form Aβ oligomers, small aggregates of 2–12 peptides, and then aggregates to form pro-fibrils and fibrils to generate the Aβ plaque (Lesné et al., 2006; Haass and Selkoe, 2007). Although Aβ deposits were for a long time thought to induce mnesic impairments, the correlation between parenchymal amyloid deposition and cognitive decline still remains controversial. Indeed, plaque density and Aβ load do not necessarily correspond to the degree of dementia. Accumulating evidence supports a detrimental role of soluble Aβ in the brains of both AD patients (Lue et al., 1999; McLean et al., 1999) and mouse models of AD (Lesné et al., 2006, 2008; Cheng et al., 2007). Such intraneuronal and extracellular accumulation of soluble Aβ oligomers may cause neuronal dysfunctions much before than neurodegeneration (Haass and Selkoe, 2007).

It is believed that AD begins with subtle alterations in the hippocampal synaptic activity prior to frank neuronal degeneration (Selkoe, 2002). Soluble Aβ may affect synaptic functions since it abrogates synaptic plasticity and induces memory impairments (Ma and Klann, 2011) and interferes with normal activity and trafficking of several synaptic receptors (Ma and Klann, 2011). Moreover Aβ is able to modify the expression of genes critical for learning and memory or neuroprotection in hippocampus (Dickey et al., 2003).

In human and mouse AD brain, microglia are associated with cerebral amyloid deposits (Dickson et al., 1988; Haga et al., 1989; Malm et al., 2005; Stalder et al., 2005; Simard et al., 2006). Microglia, the mononuclear phagocyte of the brain (Perry and Gordon, 1988), react to Aβ and have the ability to phagocytose it (Bolmont et al., 2008; Boissonneault et al., 2009; Mandrekar et al., 2009; Lee et al., 2010; Liu et al., 2010). However, the progressive Aβ accumulation together with the activation and recruitment of microglia toward plaque formation suggests that the ability of microglia to clear Aβ decreases with age and during AD progression (Hickman et al., 2008). Recent studies demonstrate that several subsets of microglia exist and can have different roles and properties (Naert and Rivest, 2011b). Microglia are replenished by division of resident cells and by recruitment of peripheral monocytes during neuropathological conditions (Simard and Rivest, 2004; Simard et al., 2006; Soulet and Rivest, 2008). Immunocompetent bone marrow-derived microglia (BMDM) have been shown to restrict amyloid pathology and prevent the cognitive decline in mouse models of AD (Simard and Rivest, 2006; Town et al., 2008; Mildner et al., 2011; Naert and Rivest, 2012). These data support the concept that microglia precursor recruitment from circulating monocytes is a natural protective mechanism against Aβ accumulation and cognitive decline.

Monocytes population is divided into three subsets in human and two subsets in mice, namely inflammatory subset CX_3_CR1^low^Ly6-C^high^Gr1^+^CCR2^+^ and the patrolling monocyte CX_3_CR1^high^Ly6-C^low^Gr1^−^CCR2^−^ (Geissmann et al., 2003; Auffray et al., 2009). Both subsets might be able of infiltrating in inflamed brain, with predominantly Ly6-C^high^ cells (Saederup et al., 2010). However, CX_3_CR1^low^Ly6-C^high^Gr1^+^CCR2^+^ subset is believed to be largely responsible of the blood-derived microglia during pathological conditions (Mildner et al., 2007; Getts et al., 2008; Naert and Rivest, 2012). The chemokine CCL2 is the main CCR2 ligand and is upregulated in brains of AD patients and mouse models of AD (Ishizuka et al., 1997; Grammas and Ovase, 2001; Simard et al., 2006; El Khoury et al., 2007; Naert and Rivest, 2011a), suggesting the preferential recruitment of CCR2^+^ monocytes. Recently we have shown that CCR2 deficiency in APP_Swe_/PS1 mice aggravated mnesic impairments and enhanced Aβ pathology (Naert and Rivest, 2011a). In addition, CCR2 deficiency specifically in bone marrow cells also enhanced AD physiopathology in APP_Swe_/PS1 mice (Mildner et al., 2011; Naert and Rivest, 2012) and these effects were prevented by the transplantation of competent CCR2 hematopoietic stem cells (Naert and Rivest, 2012). Of great interest are the data that CCR2 gene delivery in bone marrow cells rescued memory impairments of non-irradiated APP_Swe_/PS1 mice, suggesting a defect in the CCR2 system during AD (Naert and Rivest, 2012). These data underline the possibility that hematopoietic system is defective in AD subjects and in a context of APP gene overexpression.

The aim of this study was therefore to compare the immune profile together with synaptic dysfunction and soluble Aβ accumulation in APP_Swe_/PS1 mice before and after the onset of mnesic impairments. We took advantage of this mouse model since each mother delivery generates around 50% double transgenic and 50% wild-type (WT) allowing a perfect match between APP_Swe_/PS1 mice and WT littermates of the same gender. We used only male for this study to avoid the possible influence of the ovulatory cycle and variations in circulating levels of sex steroids on the immune system. Compared to their WT littermates 6-month-old APP_Swe_/PS1 mice exhibit a clear defect in the production inflammatory monocytes and this correlates with mnesic impairments, reduction in synaptic markers, and accumulation of soluble Aβ.

## Materials and methods

### Animals

We used adult male C57BL/6 mice (WT), CCR2^−/−^ (B6.129S4-Ccr2tm1Ifc/J) and APP transgenic mice (APP_Swe_/PS1) harboring the chimeric mouse/human β-amyloid precursor protein (APP695swe) and the human presenilin I (A246E variant) under the control of independent mouse prion protein promoter elements [B6C3-Tg(APP695)3Dbo Tg(PSEN1)5Dbo/J]. All the mice were originally purchased from the Jackson Laboratory and maintained in a pure C57BL/6J background. APP_Swe_/PS1 were bred with the *CCR2^−/−^* mouse strain for ≥3 generations to generate APP_Swe_/PS1/CCR2^−/−^ triple transgenic animals. All newborn pups were genotyped as described previously (Simard and Rivest, 2006) and double transgenic and WT littermates were maintained in the same environment Mice were housed 3–5 per cage and acclimated to standard laboratory conditions (12-h light, 12-h dark cycle; lights on at 07:00 and off at 19:00) with free access to mouse chow and water. Animal breeding and experiments were conducted according to Canadian Council on Animal Care guidelines, as administered by the Laval University Animal Care Committee.

### Behavioral analyses: *water T-maze*

Mice were tested during the “light-on” phase of the day. Behavioral experimenter was blind to the genetic and treatment status of animals. To assess hippocampal-dependent spatial learning and memory, mice were trained in the water T-maze task. In this paradigm, we evaluate the mouse's ability to remember the spatial location of submerged platform. The T-maze apparatus (stem length, 64 cm; arms length, 30 cm; width, 12 cm; wall height, 16 cm) was made of clear fiberglass and filled with water (23 ± 1°C) at a height of 12 cm. A platform (11 × 11 cm) was placed at the end of the target arm and was submerged 1 cm below the surface. The acquisition phase allows to evaluate animals for left-right spatial learning. During the first two trials, platforms were placed on each arm of the maze to test the spontaneous turning preference of the mouse. After these two trials, the least chosen arm was reinforced by the escape platform. The mice were placed in the stem of the T-maze and were left to choose swimming either left or right until they found the submerged platform and escaped to it for a maximum of 60 s. After reaching the platform, the mice remained on it for 20 s and were then immediately placed back into the maze. If the animals did not find the platform within this limit, they were gently guided onto it. Repeated trials were presented on the same day up to a maximum of 48 trials. A rest period of at least 10–15 min intervened between each block of 10 trials. A mouse was considered to have learned the task when it made no errors in a block of five consecutive trials. The reversal learning phase was then conducted 48 h later. During this phase, the same protocol was repeated, except that mice were trained to find the escape platform on the side opposite to where they had learned in the acquisition phase. The number of trials to reach the criterion (5/5 correct choices made on consecutive trials) was measured as well as the latency to find the escape platform.

### Tissue analyses

Mice were anesthetized under isofluorane. For first group of mice used for behavioral analysis, brains were rapidly removed from the skulls and placed for 2–4 days in 4% paraformaldehyde (PFA), pH 9.5 at 4°C, and then placed in a PFA solution containing 10% (w/v) sucrose overnight at 4°C. The frozen brains were mounted on a microtome (Reichert-Jung) and cut into 25-μm coronal sections. The slices were collected in cold cryoprotectant solution (0.05 M sodium phosphate buffer, pH 7.3, 30% ethylene glycol, and 20% glycerol) and stored at −20°C until performing immunocytochemistry or *in situ* hybridization histochemistry. A second group of mice was used for hippocampus analysis. Hippocampus were rapidly removed from the brain, frozen in liquid nitrogen, and stored at −80°C for RNA and protein analysis.

#### In situ hybridization and immunohistochemistry

Every 12th section of brain slices, starting from the end of the olfactory bulb to the end of the cerebral cortex, was mounted on Colorfrost/Plus microscope slides (Fisher Scientific) for WT and APP_Swe_/PS1 mice, from 3 to 12 months of age, (*n* = 5–12 per group). *In situ* hybridization for the histochemical localization of brain-derived neurotrophic factor (BDNF), early-growth response protein 1 (Egr1), activity-regulated cytoskeleton-associated gene (Arc), two subunits of the N-methyl-D-aspartate-type (NMDA) glutamate receptor, NR2A and NR2B, was performed using ^35^S-labeled cRNA probes. Plasmids were linearized, sense and antisense cRNA probes were synthesized with the appropriate RNA polymerase, as described in Table 1. Riboprobe synthesis and preparation and *in situ* hybridization were performed according to a previously described protocol (Laflamme et al., 1999; Laflamme and Rivest, 2001; Nadeau and Rivest, 2000; Naert et al., 2009).

**Table 1:** **Plasmids and enzymes used for the synthesis of the cRNA probes**.

**Plasmid**	**Vector**	**Insert**	**Antisense probe**	**Sense probe**	**Source**
Mouse Arc	PCR II Topo	869 bp	*EcoR I/Sp6*	*Hind III/T7*	Cloned by PCR
Mouse BDNF	PCR II Topo	342 bp	*Xho I/Sp6*	*BamH I/T7*	Dr. D. Levesque, Laval University, Québec, QC, Canada
Mouse EGR1	PCR II Topo	965 bp	*Xba I/Sp6*	*BamH I/T7*	Cloned by PCR
Mouse NR2A	PCR II Topo	1017 bp	*BamH I/SP6*	*Xho I/T7*	Cloned by PCR
Mouse NR2B	PCR II Topo	755 bp	*BamH I/T7*	*Xba I/Sp6*	Cloned by PCR

All images were captured using a Nikon Eclipse 80i microscope equipped with a digital camera (QImaging), processed to enhance contrast and sharpness using Adobe Photoshop 7 (Adobe Systems), and then assembled using Adobe Illustrator (Adobe Systems). The images depicted by the different panels are representative of the signal detected on the slides for each group of mice.

#### Stereological analysis

An observer who was blind to the treatment status of the material did all quantitative histological analyses. To count Aβ plaques, sections of 3- and 6-month-old APP_Swe_/PS1 mice (*n* = 6–15 per group) were immunostained for Aβ protein (polyclonal mouse anti-Aβ 6E10; Covariance) with 4′, 6′-diamidino-2-phenylindole, as previously reported (Simard et al., 2006; Richard et al., 2008). Four sections were chosen for hippocampus at −1.70, −1.94, −2.46, and −2.92 mm from the bregma according to a stereotaxic atlas (Paxinos and Franklin, 2nd edition). Unbiased stereological analysis was performed as described previously (Simard et al., 2006; Richard et al., 2008; Boissonneault et al., 2009). Briefly, the contours of the hippocampus areas were traced as virtual overlays on the steamed images and areas were calculated. The area occupied by all Aβ-labeled plaques was determined as previously described (Naert and Rivest, 2011a).

#### Protein and RNA extraction

Proteins from hippocampus of 3- and 6-month-old WT and APP_Swe_/PS1 mice were extracted using a modified version of the procedure published by Lesné et al. (2006). All manipulations were done on ice to minimize protein degradation. One hippocampus was placed in a 1-ml syringe with a 20 G needle. 250 μl of buffer A (50 mM Tris-HCl pH 7.6, 0.01% NP-40, 150 mM NaCl, 2 mM EDTA, 0.1% SDS, 1 mM phenylmethylsulfonyl fluoride (PMSF), protease inhibitor cocktail) were added and 10-up-and-down strokes were made to homogenize the tissue. The mixture was then separated in two tubes one for protein extraction and the other for RNA extraction.

The tube for protein was centrifuged for 5 min at 3000 rpm at 4°C. The supernatant (extracellular proteins enriched fraction) was collected and frozen at –80°C. The insoluble pellet was suspended in 250 μl TNT-buffer (Buffer B; 50 mM Tris-HCl pH 7.6, 150 mM NaCl, 0.1% Triton X-100, 1 mM PMSF, protease inhibitor cocktail), followed by a 90 min centrifugation at 13,000 rpm at 4°C. The supernatant (cytoplasmic proteins enriched fraction) was then collected and frozen at −80°C. The pellet was suspended in 250 μl buffer C (50 mM Tris-HCl pH 7.4, 150 mM NaCl, 0.5% Triton X-100, 1 mM EGTA, 3% SDS, 1% deoxycholate, 1 mM PMSF, protease inhibitor cocktail) and incubated at 4°C, 50 rpm, for 1 h. The samples were centrifuged for 90 min at 13,000 rpm and 4°C and the supernatant (membrane proteins enriched fraction) was collected and frozen at −80°C. Protein concentration of each fraction was determined using the Quantipro BCA assay kit (Sigma) according to the manufacturer protocol.

Total RNA was isolated from hippocampus mixture for 3- and 6-month-old WT and APP_Swe_/PS1 mice using TRIzol reagent method according to the manufacturer's protocol (Sigma) and then digested with deoxyribonuclease to remove any contaminating genomic DNA (Turbo DNA-free from Ambion, Austin, TX).

#### Quantitative RT-PCR analysis

RNA quantity and quality was assessed using an Agilent Technologies 2100 bioanalyzer and RNA 6000 Nano LabChip kit (Agilent, Mountain View, CA). Complementary DNA (cDNA) was generated from 40 ng of total RNA using a random primer hexamer following the protocol for Superscript II (Invitrogen, Carlsbad, CA). Equal amounts of cDNA were run in triplicate and amplified in a 15 μl reaction containing 7.5 μl of 2× Universal PCR Master Mix (Applied Biosystems, Foster City, CA), 10 nM of Z-tailed forward primer, 100 nM of reverse primer, 250 nM of Amplifluor Uniprimer probe (Chemicon, Temecula, California), and 1 μl of cDNA target. Moreover, no-template controls were used as recommended. The mixture was incubated at 50°C for 2 min, at 95°C for 4 min, and then cycled at 95°C for 15 s and at 55°C for 30 s, 55 times using the Applied Biosystems Prism 7900 Sequence Detector. Amplification efficiencies were validated and normalized to ribosomal 18S gene and quantity of target gene was calculated according to a standard curve. Primers sequences were designed using Primer Express 2.0 (Applied Biosystems) and are described in Table 2. Amplicons were detected using the Amplifluor UniPrimer system, in which forward primers used contained a 5′ Z sequence: ACTGAACCTGACCGTACA.

**Table 2:** **Primers sequence for real time PCR**.

**Gene (*mus musculus*)**	**mRNA RefSeq number**		**Primer sequence 5′ → 3′ (Z-tail)**
Arc	NM_018790	Fwd	ACTGAACCTGACCGTACAAGGGTGAACCACTCGACCAGT
		Rev	TCTGGTACAGGTCCCGCTTG
Bdnf	NM_001048142	Fwd	CTGACGACGACATCACTGGC
		Rev	ACTGAACCTGACCGTACACAGCTCTTCGATGACGTGCTC
Dlg4 (PSD95)	NM_001109752	Fwd	ATTCCCAGCAAACGGCG
		Rev	ACTGAACCTGACCGTACAGGCCTTTAACCTTGACCACTCTC
Egr1	NM_007913	Fwd	ACTGAACCTGACCGTACAAGCACCTGACCACAGAGTCCTT
		Rev	CCTTCTCATTATTCAGAGCGATGTC
Grin2a (NR2A)	NM_008170	Fwd	ACTGAACCTGACCGTACATAGCAGGCCCTCTCGTAGCA
		Rev	TCCAGTAGCCTTTCCCTGTCC
Grin2b (NR2B)	NM_008171	Fwd	TATGTGGACCAGGTTTCCGG
		Rev	ACTGAACCTGACCGTACAATTAGGTCTCTGGAACTTCTTGTCACT
Gria1 (AMPA 1)	NM_008165	Fwd	ACTGAACCTGACCGTACATGCCAATTTCCCCAACAATATC
		Rev	TGTTGGTTTGGAAATAATCCCC
h18S/m18S	M10098	Fwd	ACTGAACCTGACCGTACACGGTACAGTGAAACTGCGAATG
		Rev	CCAAAGGAACCATAACTGATTTAATGA
Ntrk2 (TrkB full length)	NM_008745	Fwd	GTGGTGTCATTAGTAGGTTCTTTGTTTT
		Rev	ACTGAACCTGACCGTACAGAGTTTGGGTCTTTGCTGCC
Ntrk2 (TrkB T1)	NM_001025074	Fwd	GACCTCAACAAGTTCCTTAGGGC
		Rev	ACTGAACCTGACCGTACATGCCATCAGCACTGCGTC
Ntrk2 (TrkB T2)	BC052014	Fwd	CCCAGTTGGTGGTGTGTGG
		Rev	ACTGAACCTGACCGTACAGGACAAACAGACATGGATGAGACT

#### Detection of total Aβ levels by Western blot

For total Aβ detection, 10–20 μg of extracellular, cytoplasmic, and membrane protein fractions of hippocampus were separated on a precast 4–20% polyacrylamide Tris-Glycine eXtended (TGX) gel (Bio-Rad). Separated proteins were then transferred onto polyvinylidene fluoride (PVDF) membranes (PerkinElmer Life and Analytical Sciences) and detected by Western blotting. Blots were probed with a mouse anti-amyloid beta protein monoclonal antibody clone 6E10 (1:1500, Covariance) in 1 M Tris-HCl, pH 8.0, 5 M NaCl, 5% skim milk, and 0.05% Tween 20. Blots were visualized using anti-mouse secondary antibody tagged with horseradish peroxidase (1:1000; Jackson Immuno-Research) and enhanced chemiluminescence (PerkinElmer Life and Analytical Sciences). Membranes were stripped in 25 mM glycine-HCl, pH 2.0, containing 1% SDS to allow β-actin revelation using first a mouse β-actin antibody (MAB1501, 1:5000; Millipore Bioscience Research Reagents) and then a goat anti-mouse peroxidase conjugated secondary antibody (1:1000; Jackson ImmunoResearch).

Quantification was done by determining integrative density of the bands using a gel imaging system (scanner Agfa Arcus II; NIH Image J software version 1.32j) and background values were removed. Optical values were normalized according to the actin loading control. Results are expressed as mean ± SEM.

### FACS analyses

#### Analysis of circulating monocytes

Fluorescence-activated cell sorting (FACS) analysis was performed on red blood cell-lysed blood of 5-week- and 6-month-old WT and APP_Swe_/PS1 mice. Blood of CCR2^−/−^ mice was used as negative control of CCR2 staining. To analyze the population of monocytes, anti-coagulated whole blood was taken from the facial vein and quickly suspended and cells were washed in Dulbecco's PBS (DPBS) + 4% fetal bovine serum (FBS). Cells, suspended in DPBS + 2% FBS, were first incubated on ice for 15 min with purified rat anti-mouse CD16/CD32 (Mouse BD Fc Bloc, BD Bioscience). The mix was then incubated on ice with Phycoerythrin (PE)-conjugated CD45 antibody (BD Bioscience), PE-Cy7™-conjugated CD11b antibody (eBioscience), allophycocyanin (APC)-conjugated CD115 antibody (eBioscience), FITC-conjugated Ly6-C antibody (BD Bioscience), and PerCP-Cy5.5™-conjugated Gr1 antibody (Cedarlane) for 35 min. Cells were washed again in DPBS + 2% FBS. Red blood cells were lysed with hemolysin according to the manufacturer's protocol (Beckman Coulter, Mervue, Galway, Ireland) and cells were washed with DPBS and re-suspended in equal volumes of DPBS. For CCR2 detection, washed cells were first incubated on ice with monoclonal antibody MC-21 (anti-CCR2) for 60 min (Mack et al., 2001). After washing, cells were incubated for 60 min on ice with a biotin-labeled anti-rat polyclonal antibody (BD Bioscience). Cells were washed again and incubated with purified rat anti-mouse CD16/CD32 on ice for 15 min, before adding PerCP™-labeled streptavidin (BD Bioscience) and the combination of directly conjugated antibodies as described previously (Mack et al., 2001). Cells were analyzed using a two-lasers and six color FACS Canto II flow cytometer and data acquisition was done with BD Facs Diva software (version 6.1.2, BD Biosciences, Mississauga, ON, Canada). Cells were then sorted according to the different fluorescent antibodies. Results were analyzed using Flow Jo software (Tristar).

#### Determination of Ly6-C^high^ monocytes in the bone marrow

Male WT and APP_Swe_/PS1 mice, 6–6.5 months of age, were used to determine the frequency of inflammatory monocytes in the bone marrow. The cells were aseptically harvested for each mice by flushing the two tibias and femurs with DPBS containing 2% FBS. Cell samples were combined for each mouse, filtered through a 40-μm nylon mesh, centrifuged, and were washed with DPBS and re-suspended in equal volumes of DPBS + 2% FBS. Cells were first incubated on ice for 15 min with purified rat anti-mouse CD16/CD32 (Mouse BD Fc Bloc, BD Bioscience). The mix was then incubated on ice with PE-conjugated CD11b antibody (BD Bioscience), horizon V500-conjugated CD45 antibody (BD Bioscience), and Pacific blue-conjugated Ly6-C antibody (Biolegend) for 35 min. Cells were analyzed using a two-lasers and six color FACS Canto II flow cytometer and data acquisition was done with BD Facs Diva software (version 6.1.2, BD Biosciences, Mississauga, ON, Canada). Cells were then sorted according to the different fluorescent antibodies. Results were analyzed using Flow Jo software (Tristar).

### Macrophage colony-stimulating factor (M-CSF) treatment

WT and APP_Swe_/PS1 mice received daily an ip injection of murine M-CSF (40 μg/Kg) (R&D Systems) during 4 days at the age of 3 or 6 months and were tested 3 months later in the T-maze paradigm. The experimental procedure for these two protocols is depicted in the Figure 8. The first experiment consisted to treat animals before the occurrence of mnesic impairments and Aβ pathology (Figure 8A), whereas in the second experiment animals were administered after the occurrence of cognitive decline, synaptic disruption and Aβ pathology (Figure 8D).

To determine the potential role of CCR2 in mediating the effects of M-CSF on monocytes, WT, CCR2, APP_Swe_/PS1, and APP_Swe_/PS1/CCR2^−/−^ mice received daily ip injections of the cytokine (40 μg/Kg) during 4 days at the age of 3.5–4 months. Twenty-four hours after the fourth injection, the whole blood was taken from the facial vein to perform FACS analysis. As previously explained, PE-Cy7™-conjugated CD11b antibody (eBioscience), PE-conjugated CD45 antibody (BD Bioscience), and APC-conjugated CD115 antibody (eBioscience) were used to determine the population of monocytes.

### Statistical analyses

Results are expressed as the mean ± SEM. Statistical analysis was performed by one- or two-way analysis of variance (ANOVA), followed by the appropriate test procedure, using Bonferroni or Tamhane's tests as *post-hoc* comparisons (SPSS software). Data were analyzed using standard two-tailed unpaired *t*-test's for the comparison between two groups. Correlations were estimated by linear regression analysis and the Spearman's correlation coefficient (GraphPad Software Inc.) with *p*- and *r*-values and 95% confidence intervals included in the graph. A *p*-value <0.05 was considered statistically significant.

## Results

### The occurrence of mnesic impairments does not correlate with Aβ deposits

**Figure 1:**
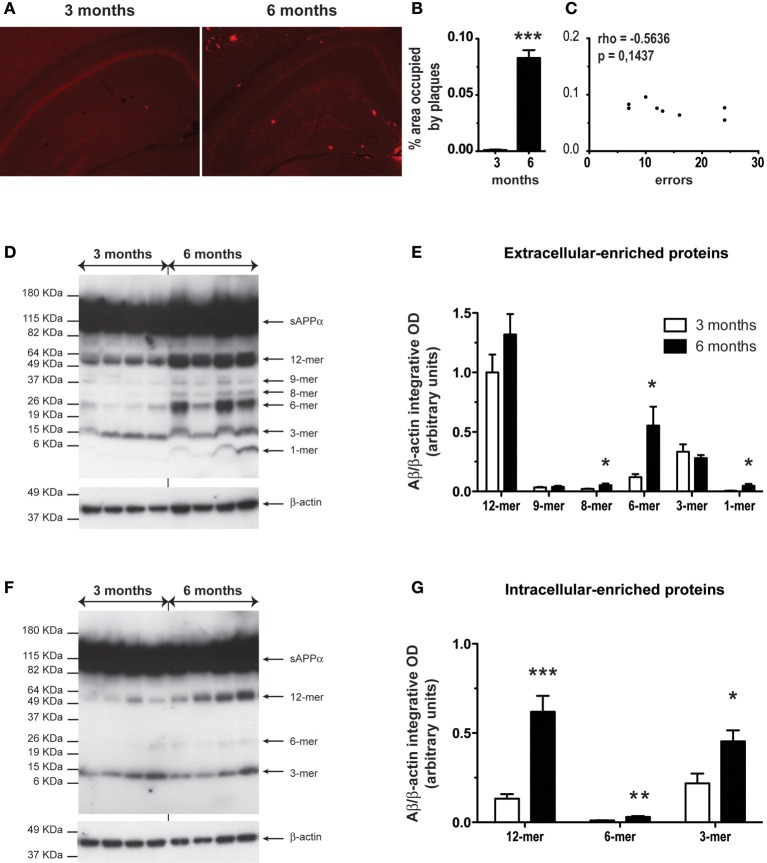
**Six-month-old APP_Swe_/PS1 mice had higher level of Aβ soluble oligomers in the hippocampus**. Anti-Aβ immunoreactivity is depicted in hippocampus of APP_Swe_/PS1 at 3 and 6 months of age **(A)**. A detailed analysis of plaque quantification was performed to determine the percentage area occupied by plaques **(B)** of 3- and 6-month-old APP_Swe_/PS1. Aβ load was strongly increased at 6 months age in hippocampus. A correlation's test revealed no significant correlation between spatial memory decline (water T-maze test) and the percentage area occupied by plaques at the age of mnesic deficit occurrence (6 months) **(C)**. Therefore, the hippocampus level of soluble Aβ was analyzed by western blotting for extracellular and intracellular-associated proteins of 3- and 6-month-old APP_Swe_/PS1 mice. The intensity of each band was quantified by densitometric analysis and normalized per β-actin values. Aβ species ratios (Aβ/β-actin) are represented and increased levels of Aβ soluble oligomers were observed at 6 months of age in extracellular **(D–E)** and intracellular **(F–G)**. Results are expressed as the Mean ± SEM; *n* = 6–7; Student's *t*-test; ^*^p < 0.05, ^**^p < 0.01 and ^***^p < 0.001. ^*^ vs. APP_Swe_/PS1 at 3 months of age. Magnification 4×. Correlation test was performed using the Spearman's correlation coefficient.

Coronal brain sections of WT and APP_Swe_/PS1 mice at 3 and 6 months of age were immunostained with the 6E10 antibody specific for the 1–16 fragment of the human Aβ protein. Plaques surface were determined by stereological analysis in hippocampus. Aβ deposits are scarce in 3-month-old APP_Swe_/PS1 mice (Figures 1A–B). Indeed, insoluble Aβ begins to accumulate at 6 months of age (Figures 1A–B). Density and area occupied by Aβ deposits are robustly increased in hippocampus at 6 months of age (Figures 1A–B). These results seem to support a positive correlation between the onset of plaque formation and cognitive impairment. Therefore, we investigated if mnesic deficit correlated positively with Aβ load at the age of 6 months. We determined the correlation between spatial memory decline (water T-maze test) and the percentage area occupied by plaque in hippocampus in each 6-month-old APP_Swe_/PS1 mice. Surprisingly no correlation was observed for both structures (Figure 1C).

As mnesic impairments occurred at 6 months of age (Naert and Rivest, 2011a), we precisely compared the hippocampal level of soluble Aβ oligomers in APP_Swe_/PS1 mice at 3 and 6 months of age. Using a modified version of the Lesné's protocol (Lesné et al., 2006), we performed western blotting for extracellular- and intracellular-enriched proteins. The different Aβ oligomers were revealed using the 6E10 antibody. In the extracellular-enriched fraction, 6-month-old APP_Swe_/PS1 mice exhibited an important increase of low-n oligomers. Indeed levels of 8-, 6-, and 1-mer were significantly increased (Figures 1D–E). In the intracellular fraction, soluble oligomers (12-, 6-, and 3 -mer) were augmented (Figures 1F–G). Therefore, a robust accumulation of soluble Aβ occurred in hippocampus of 6-month-old APP_Swe_/PS1 mice.

### APP_Swe_/PS1 mice at the age of 6 months exhibited a decrease in transcript level of BDNF and its receptors in hippocampus

**Figure 2:**
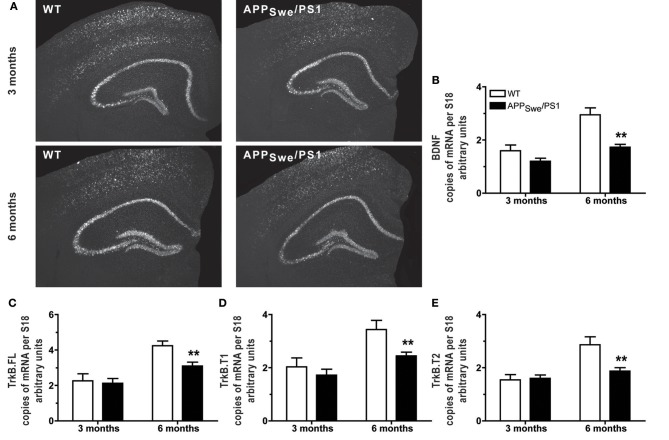
**Expression of BDNF and its receptors decreased in hippocampus when mnesic deficits begin in APP_Swe_/PS1 mice**. Representative dark-field photomicrographs of *in situ* hybridization showed the hippocampal expression of BDNF mRNA **(A)** in the brain of 3- and 6-month-old WT and APP_Swe_/PS1 mice. For hippocampus, mRNA levels of BDNF and its receptors—TrkB.FL, TrkB.T1, and TrkB.T2—were determined by real-time qPCR and normalized relative to the level of S18 mRNA detected in each sample. At 3 months of age, APP_Swe_/PS1 mice exhibited similar mRNA levels of BDNF **(A** and **B)** and its receptors, TrkB.FL **(C)**, TrkB.T1 **(D),** and TrkB.T2 **(E)**. In contrast, all transcripts were significantly decreased in 6 month-old APP_Swe_/PS1 mice compare to WT littermates **(B–E)**. Results are expressed as the Mean ± SEM; *n* = 5–9; Student's *t*-test; ^**^p < 0.01 vs. WT at the same age.

As mnesic impairment appeared at the age of 6 months in APP_Swe_/PS1 mice, we compared the BDNF transcript levels in WT and APP_Swe_/PS1 mice at 3 and 6 months of age. WT and APP_Swe_/PS1 mice exhibited similar expression of BDNF mRNA in hippocampus at 3 months of age, as revealed by hybridization *in situ* (Figure 2A) and quantitative RT-PCR analysis (Figure 2B). On the other hand, 6-month-old APP_Swe_/PS1 mice had lower BDNF mRNA levels in hippocampus, a 41% reduction was highlighted by quantitative RT-PCR analysis (Figure 2B). BDNF activity is dependent on TrkB and its downstream targets and levels of the different isoforms of TrkB—TrkB full length (TrkB.FL), TrkB T1, and TrkB T2—were assessed by quantitative RT-PCR analysis in hippocampus. WT and APP_Swe_/PS1 mice expressed similar TrkB.FL, TrkB T1, and TrkB T2 mRNA levels at 3 months of age (Figures 2C–E), whereas these transcript levels were significantly reduced by 27, 29, and 35%, respectively, in 6-month-old APP_Swe_/PS1 mice (Figures 2C–E). These data suggest that the BDNF system is affected in the in hippocampus of 6-month-old APP_Swe_/PS1 mice.

### Decreased expression of genes encoding synaptic proteins that are critical for memory and learning in the hippocampus of 6-month-old APP_Swe_/PS1 mice

**Figure 3:**
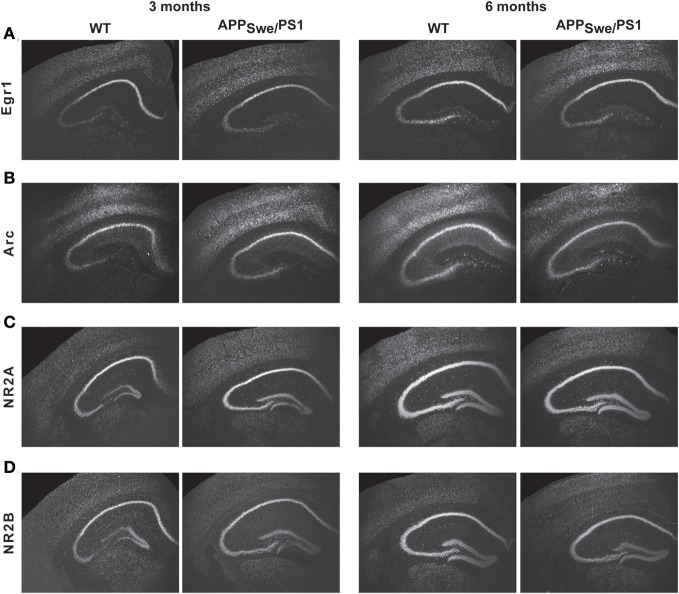
**Egr1, Arc, NR2A, and NR2B transcript levels in APP_Swe_/PS1 mice**. Expression of Egr1, Arc, PSD95, AMPA1, NR2A, and NR2B mRNA were determined by *in situ* hybridization. Three-month-old WT and APP_Swe_/PS1 mice had similar mRNA levels of Egr1 **(A)**, Arc **(B)**, NR2A **(C)**, and NR2B **(D)**. In contrast, intensity of transcript signal for Egr1 **(A)**, Arc **(B)**, NR2A **(C)**, and NR2B **(D)** was lower in the brain of APP_Swe_/PS1 mice than WT mice at 6 months of age. Magnification 4×.

**Figure 4:**
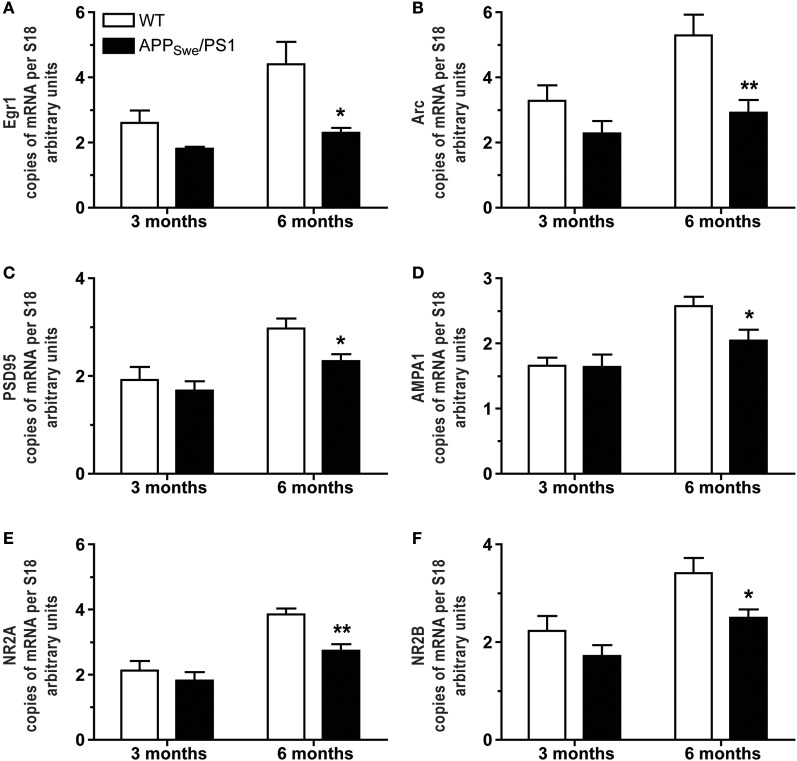
**Occurrence of mnesic deficits correlate with decreased mRNA levels of synaptic markers in hippocampus of APP_Swe_/PS1 mice**. Real-time qPCR was used to quantify Egr1, Arc, PSD95, AMPA1, NR2A, and NR2B mRNA levels in the hippocampus. The values were normalized relative to the level of S18 mRNA detected in each sample. 3-month-old WT and APP_Swe_/PS1 mice had similar mRNA levels of Egr1 **(A)**, Arc **(B)**, PSD95 **(C)**, AMPA1 **(D)**, NR2A **(E)**, and NR2B **(F)**. In contrast, all transcripts were significantly decreased in APP_Swe_/PS1 mice at 6 months of age when mnesic deficits begin. Results are expressed as the Mean ± SEM; *n* = 5–9; Student's *t*-test; ^*^p < 0.05 and ^**^p < 0.01 vs. WT at the same age.

Expression of pre- and post-synaptic genes was assessed in hippocampus by *in situ* hybridization and RT-PCR. We performed *in situ* hybridization for Egr1, Arc, NMDA-R1 (NR2A), and -R2 (NR2B) transcripts in hippocampus of 3- and 6-month-old WT and APP_Swe_/PS1 mice (Figure 3). At 3 months of age, WT and APP_Swe_/PS1 mice exhibited similar signal for all transcripts, whereas a decreased signal for Egr1, Arc, NR2A, and NR2B mRNA was observed in the hippocampus of 6-month-old APP_Swe_/PS1 mice (Figure 3). These results were confirmed using RT-PCR analysis (Figure 4). Similar expression levels of these transcripts were quantified in the hippocampus at 3 months of age. However, Egr1, Arc, NR2A and NR2B, AMPA1, and PSD95 mRNA levels were significantly lower by 48, 47, 29, 27, 21, and 22%, respectively, in APP_Swe_/PS1 mice then their WT littermates (Figure 4). These data highlight a defect in synaptic markers in hippocampus of 6-month-old APP_Swe_/PS1 mice.

### Frequency of Ly6-C^high^GR1^+^CCR2^+^ monocytes is significantly reduced in the bloodstream and the bone marrow of 6-month-old APP_Swe_/PS1 mice

**Figure 5:**
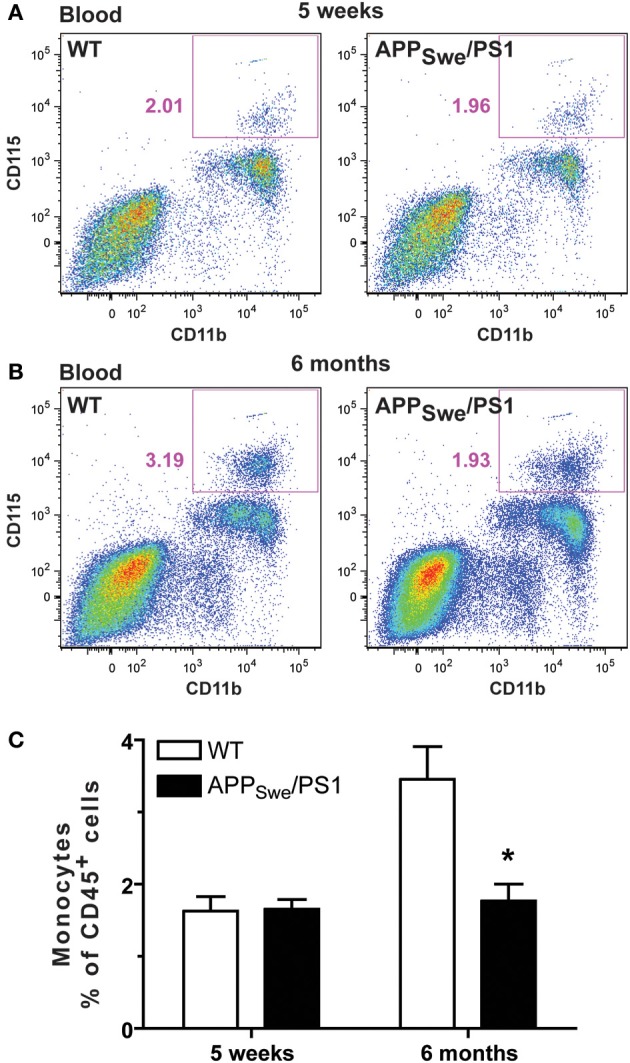
**Monocyte levels are drastically diminished in 6-month-old APP_Swe_/PS1 mice**. Circulating monocyte numbers were determined by FACS analysis within the population of leukocytes (CD45^+^) in the blood of WT and APP_Swe_/PS1 mice at 5 weeks and 6 months of age. Monocytes were characterized by CD11b and CD115 expression and were quantified within the population of CD45^+^ cells. At 5 weeks of age, WT and APP_Swe_/PS1 mice exhibited similar levels of monocytes **(A** and **C)**. In contrast, APP_Swe_/PS1 mice had fewer monocytes than WT mice at 6 months of age **(B** and **C)**. Results are expressed as the Mean ± SEM; *n* = 7–8; Student's *t*-test; ^*^p < 0.05; ^*^ vs. WT at the same age.

To assess whether monocytes were affected in a context of AD and whether the occurrence of memory deficit may be linked with impairment of the innate immune system, we determined the frequency of monocytes in WT and APP_Swe_/PS1 mice before (5 weeks of age) and at the beginning of the mnesic deficit (6 months of age). Leukocytes were labeled with CD45 and monocytes were distinguished using CD11b and CD115 (CSF1/MCSF receptor). At 5 weeks WT and APP_Swe_/PS1 mice exhibited similar frequency of CD45^+^CD11b^+^CD115^+^ cells (Figures 5A and C) and this monocyte number significantly increased in 6-month-old WT mice as depicted in the Figure 5C. Such a phenomenon has previously been described (Boggs et al., 1986). Of great interest is that monocyte frequency did not increase in 6-month-old APP_Swe_/PS1 mice and their circulating monocyte number remained at the level of the young 5 week-old animals (Figures 5A–C).

**Figure 6:**
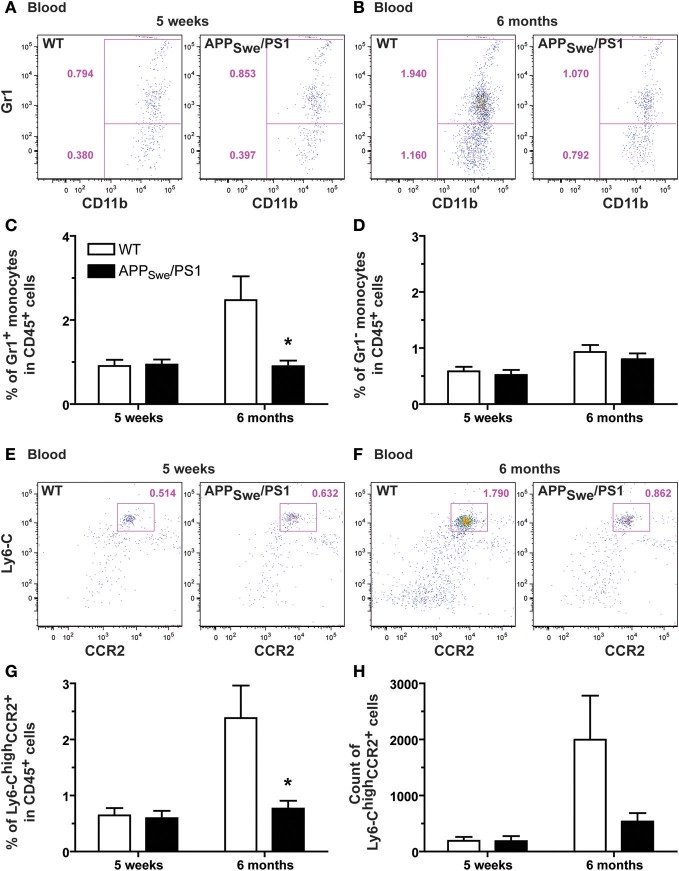
**Defective production of Gr1^+^Ly6-C^high^CCR2^+^ monocytes in 6-month-old APP_Swe_/PS1 mice**. *Gr1^+^Ly6-C^high^CCR2^+^* monocytes were quantified by FACS analysis in the blood of WT and APP_Swe_/PS1 mice at 5 weeks and 6 months of age. Monocytes were determined by the presence of CD11b and CD115. Different subsets of monocytes were characterized using Gr1, Ly6-C, and CCR2 antibodies. At 5 weeks of age, WT and APP_Swe_/PS1 mice exhibited similar levels of Gr1^+^ **(A** and **C)** and Gr1^−^ monocytes **(A** and **D)**. A marked increase in the population of Gr1^+^ monocytes was found in the bloodstream of 6-month-old WT mice, but this phenomenon was totally prevented in APP_Swe_/PS1 mice **(B** and **C)**. Although these mice failed to exhibit such increase in Gr1^+^ monocytes, they had similar number of blood Gr1^−^ monocytes when compared to WT animals **(B** and **D)**. The low frequency of Gr1^+^Ly6-C^high^CCR2^+^ monocytes was confirmed in 6-month-old APP_Swe_/PS1 mice using more specific markers of the Gr1^+^ monocyte (e.g., Ly6-C and CCR2) **(E–H)**. Results are expressed as the Mean ± SEM; *n* = 7–8; Student's *t*-test; ^*^p < 0.05; ^*^ vs. WT at the same age.

We then distinguished between the inflammatory subset CX_3_CR1^low^Ly6-C^high^Gr1^+^CCR2^+^ and the patrolling monocyte CX_3_CR1^high^Ly6-C^low^Gr1^−^CCR2^−^ by the expression of Gr1 (Figure 6). At 5 weeks of age, WT and APP_Swe_/PS1 mice exhibited similar frequency of each subset (Figures 6A–D). The ratio of Gr1^+^ and Gr1^−^ monocyte is ~60% and ~40%, respectively, in both group of mice (Figures 6A–D). At 6 months of age the frequency of Gr1^−^ monocytes was similar between WT and APP_Swe_/PS1 mice (Figures 6B and D), but the proportion of Gr1^+^ is reduced by almost 3 fold in 6-month-old APP_Swe_/PS1 mice compared to their WT littermates (Figures 6A and C). This group of WT mice had ~70% of Gr1^+^ and ~30% Gr1^−^ monocyte, which is 2 fold more Gr1^+^ subset than their APP_Swe_/PS1 progeny (Figures 6C and D). To confirm these results we used Ly6-C and CCR2 to further distinguish between the different subsets of monocytes. Blood of CCR2-deficient mice was used as negative control for the CCR2 antibody used in this study (data not shown). According to the previous observation, we found a marked decrease of Ly6-C^high^CCR2^+^ monocytes in 6-month-old APP_Swe_/PS1 mice (Figures 6G–H), while Ly6-C^int^ and Ly6-C^low^ monocytes remained similar in WT and APP_Swe_/PS1 mice at the same age (data not shown).

**Figure 7:**
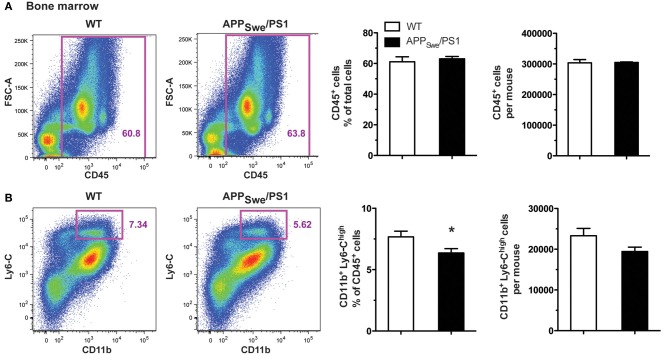
**Gr1^+^Ly6-C^high^CCR2^+^ monocyte frequency is diminished in the bone marrow of 6-month-old APP_Swe_/PS1 mice**. Leukocytes and Gr1^+^Ly6-C^high^CCR2^+^ monocytes were quantified by FACS analysis in the bone marrow of WT and APP_Swe_/PS1 mice at 6 months of age. The leukocyte population was assessed using the CD45 marker. At 6 months of age, WT and APP_Swe_/PS1 mice exhibited similar levels of leukocytes **(A)**. In the population of CD45^+^ cells, the inflammatory monocytes were determined by the presence of CD11b and high expression levels of Ly6-C. A marked decrease in the population of Ly6-C^high^ monocytes was found in the bone marrow of 6-month-old APP_Swe_/PS1 mice **(B)**. Results are expressed as the Mean ± SEM; *n* = 6–7; Student's *t*-test; ^*^p < 0.05; ^*^ vs. WT.

To explain the defect in circulating Ly6-C^high^ monocytes, we assessed the frequency of this monocyte subset in the bone marrow of 6-month-old APP_Swe_/PS1 mice. Using the common leukocyte antigen CD45, we found a similar frequency and number of CD45^+^ cells in both WT and APP_Swe_/PS1 groups of mice at 6 months of age (Figure 7A). CD11b and Ly6-C were consequently used to identify the inflammatory monocyte subset among these CD45^+^ cells and APP_Swe_/PS1 mice had significantly less Ly6-C^high^ monocytes in their bone marrows than WT littermates (Figure 7B). This interesting result suggests strongly that the production of Ly6-C^high^ monocytes is decreased in the bone marrow of 6-month-old APP_Swe_/PS1 mice. There is consequently a clear defect of the hematopoietic system to produce Ly6-C^high^CCR2^+^ monocytes in 6-month-old APP_Swe_/PS1 mice and this correlates with the beginning of the cognitive decline of this mouse model of AD.

### A short macrophage colony-stimulating factor (M-CSF) treatment increases monocyte frequency in APP_Swe_/PS1 mice and rescues their cognitive impairment

**Figure 8:**
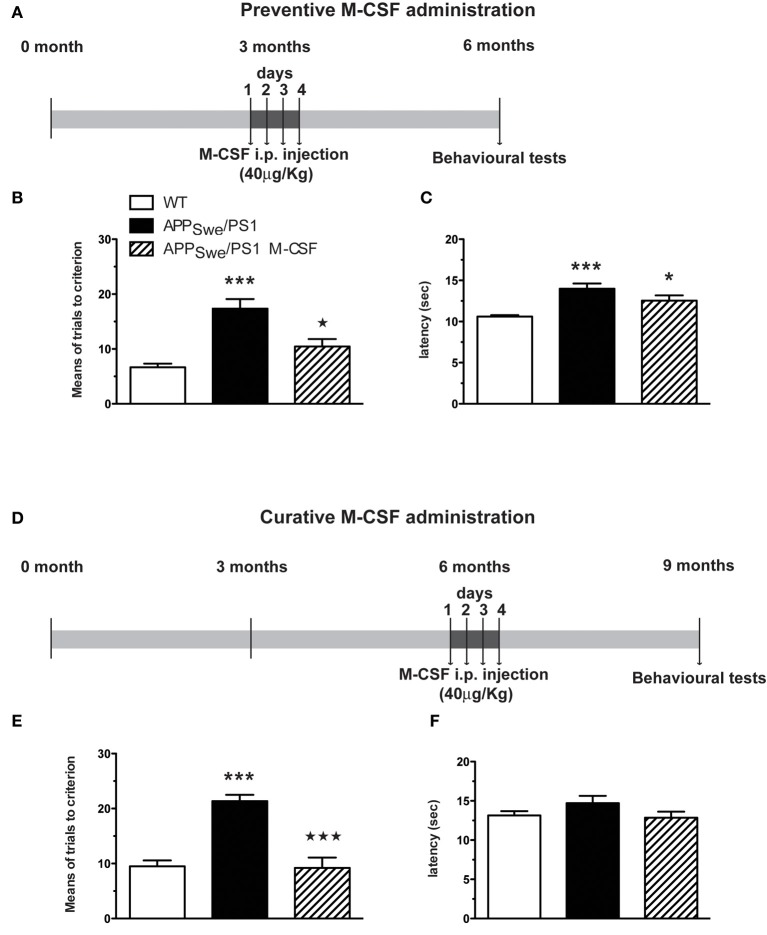
**Short M-CSF treatment is able to prevent and to rescue learning and memory impairment in APP_Swe_/PS1 mice**. APP_Swe_/PS1 mice received M-CSF treatment during 4 days (40 μg/Kg/day) before (at 3 months) or after occurrence of mnesic deficit (at 6 months) and were tested in the T water-maze paradigm 3 months later **(A)**. The numbers of trials **(B** and **E)** and the latency **(C** and **F)** to accomplish the task were determined during reversal learning phase. M-CSF treatment prevented spatial memory decline generally observed in 6-month-old APP_Swe_/PS1 mice **(B)**. In addition, M-CSF treatment after occurrence of mnesic impairment (at 6 months) **(D)** rescued spatial memory in 9-month-old APP_Swe_/PS1 mice **(E)**. Results are expressed as the Mean ± SEM; *n* = 8–16 per group; ^*^p < 0.05 and ^***^p < 0.001; ^*^ vs. WT, ^⋆^ vs. APP_Swe_/PS1 mice. (One-way ANOVA was performed in each genotype using Bonferroni or Tamhane's *post-hoc* test).

To assess whether defect in the population of monocytes is due to their inability to differentiate properly and is a non-reversible mechanism, we assessed the effect of the main hematopoietic cytokine involved in monocytopoeisis, M-CSF, in APP_Swe_/PS1 mice. A short treatment with M-CSF (once/day, during 4 days) was administered in APP_Swe_/PS1 mice before (at 3 months) and after the occurrence of cognitive decline (at 6 months). Spatial memory of these mice was assessed 3 months later using the water T-maze test (Figures 8A and D). M-CSF was able to prevent the apparition of memory impairment, which occurred normally at 6 months, as revealed by the number of trials (10.4 ± 1.4 vs. 17.4 ± 1.7 for APP_Swe_/PS1 mice; *p* < 0.05) (Figures 8A–C). M-CSF did also restore mnesic capacity—M-CSF-treated APP_Swe_/PS1 mice at 9 months of age exhibited a significant decrease of the number of trials (9.2 ± 1.9 vs. 21.4 ± 1.1 for APP_Swe_/PS1 mice; *p* < 0.001) (Figures 8D–F).

**Figure 9:**
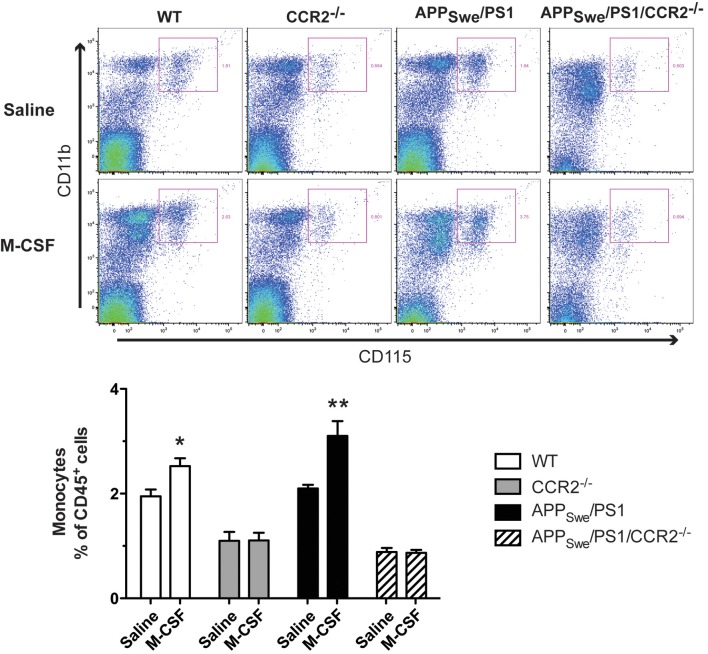
**Short M-CSF treatment increases monocyte frequency in WT and APP_Swe_/PS1 mice in a CCR2-dependent manner**. Monocyte frequency was determined by FACS analysis within the population of CD45^+^ cells using the CD11b and CD115 markers in WT, CCR2^−/−^, APP_Swe_/PS1, and APP_Swe_/PS1/CCR2^−/−^ mice. Mice received saline or M-CSF treatment during 4 days (40 μg/Kg/day) at the age of 4 months and blood samples were analyzed 24 h after the last injection. M-CSF treatment increased the frequency of monocytes (CD11b^+^CD115^+^) in both WT and APP_Swe_/PS1 groups of mice. In contrast, monocyte frequency remained very low in CCR2^−/−^ and APP_Swe_/PS1/CCR2^−/−^ mice treated or not with the cytokine. Results are expressed as the Mean ± SEM; *n* = 5–8; Student's *t*-test; ^*^p < 0.05; ^**^p < 0.01; ^*^ vs. saline treatment in the same genotype.

To determine whether M-CSF had the same effect on monocytic commitment in WT and APP_Swe_/PS1 mice, we performed FACS analysis on blood cells 24 h after the fourth injection of M-CSF (40 μg/Kg/day). Such a treatment increased the number of monocytes (CD11b^+^CD115^+^) within the population of CD45^+^ cells (Figure 9). M-CSF caused a similar increase of monocyte frequency in APP_Swe_/PS1 mice (Figure 9). This effect of M-CSF was dependent on CCR2, because the cytokine failed to increase the population of monocytes in CCR2^−/−^ and APP_Swe_/PS1/CCR2^−/−^ mice (Figure 9).

## Discussion

Immune system is a key feature in AD pathology, but its role has yet to be fully unraveled. Here, we have analyzed monocyte subset frequency associated to age-dependent amyloid pathological progression in APP_Swe_/PS1 mice. The main findings are the reduced levels of monocytes, due to the decreased frequency of CX_3_CR1^low^Ly6-C^high^Gr1^+^CCR2^+^ subset in bloodstream and bone marrow of 6-month-old APP_Swe_/PS1 mice. This defect in monocyte levels corresponds to the onset of memory impairment, synaptic disruption, down-regulation of neuroprotective factor, and an enhanced accumulation of soluble Aβ in hippocampus.

While density and area of Aβ plaques do not closely correspond to the degree of dementia, increased levels of soluble Aβ seem more closely associated with cognitive decline in AD (Lue et al., 1999; McLean et al., 1999; Naslund et al., 2000; Lesné et al., 2006, 2008; Cheng et al., 2007). As we previously observed in others mouse models of AD (Richard et al., 2008; Naert and Rivest, 2011a, 2012), neither onset nor intensity of the memory decline correlated with the senile plaques at all ages studied in APP_Swe_/PS1 mice. A significant increase of intra- and extracellular pools of Aβ was found in hippocampus of 6-month-old APP_Swe_/PS1 mice. This increase occurred concomitantly with the apparition of mnesic impairments and the deficit of synaptic markers. Soluble Aβ was detected before plaque formation, synaptic impairments, neurotrophin deficits, and memory decline (at 3 months of age). In the Tg2576 model, memory impairment begins 6 months before plaque formation when Aβ dimers start to accumulate (Kawarabayashi et al., 2004). Indeed soluble Aβ can induce cognitive impairment (Larson and Lesné, 2011), such as extracellular Aβ*56 (Lesné et al., 2006) or soluble Aβ dimer and trimer (Cleary et al., 2005; Shankar et al., 2008). In AD patients, soluble intracellular and membrane-associated Aβ in temporal neocortex is more closely related to AD symptoms than other Aβ species (Steinerman et al., 2008). Increased levels of intraneuronal Aβ aggravated the mnesic deficit in APP_Swe_/PS1/CCR2^−/−^ mice (Naert and Rivest, 2011a), suggesting that accumulation of soluble intraneuronal Aβ is a key feature for the synaptic pathology in AD.

Synaptic plasticity is involved in learning and memory, and requires the expression of proteins able to mediate changes necessary for the increase in synaptic strength and memory. Alterations in synapses appear to be among the earliest events in the initiation of the cognitive decline that characterize AD and have long been considered the best pathological correlates of cognitive decline in AD (Coleman and Yao, 2003). Correlations between alterations in BDNF expression and/or function and mechanisms occurring in AD are well established (Connor et al., 1997; Tapia-Arancibia et al., 2008). Here we also observed a strong decrease of BDNF mRNA in hippocampus of 6-month-old APP_Swe_/PS1 mice. At the same time the different isoforms of its specific receptors, TrkB—TrkB FL, T1, and T2—are all down-regulated. Expression of BDNF is significantly reduced in AD and mildly cognitively impaired patients (Tapia-Arancibia et al., 2008). Recently, impaired TrkB receptor signaling was reported to contribute to memory impairments in APP_Swe_/PS1 dE9 mice (Kemppainen et al., 2012). The *BDNF* gene delivered into amyloid-transgenic mice was found to reverse synapse loss, resulting in partial recovery of synaptic markers, and restoration of learning and memory (Nagahara et al., 2009).

In addition the immediate early genes Arc and Egr1, which are clearly involved in learning and memory processes in hippocampus, were drastically reduced in 6-month-old APP_Swe_/PS1 mice. Subunits of ionotropic glutamate receptors, NMDA (NR2A and NR2B) and AMPA (AMPA1), and PSD95 were also significantly decreased in the hippocampus of these mice. Overexpression of the NR2B subunit leads to improved memory function (Tang et al., 1999), whereas hippocampal injection of antisense oligonucleotides directed against NR2B mRNA inhibits cognitive functions (Clayton et al., 2002). These molecules (e.g., AMPA1 and PSD-95) are critical for synaptic plasticity (Malinow and Malenka, 2002). PSD-95 is involved in recruiting and holding glutamate receptors at the surface (Xiao et al., 1998; Ango et al., 2000) and Arc expression is also closely related to NMDA activity (Bloomer et al., 2008). BDNF stimulates Arc expression in neurons by a mechanism that involves the TrkB receptor and is also dependent on the NMDA glutamate receptor (Yin et al., 2002). BDNF plays a key role in regulating expression and synaptic delivery of AMPA receptor subunits (Li and Keifer, 2009) and it regulates the synaptic localization of PSD95 via a TrkB-dependent mechanism (Yoshii et al., 2011) and enhances AMPA trafficking to cell membrane (Li and Keifer, 2009).

Down-regulated expression of these genes has a clear impact on synaptic plasticity processes underlying long-term potentiation and memory in AD. This decrease of synaptic genes can be explained by the accumulation of soluble Aβ since hippocampus of 6-month-old APP_Swe_/PS1 mice had elevated levels of both extracellular and intracellular Aβ. Both intra- and extracellular Aβ has the ability to affect expression of learning related genes (Wegenast-Braun et al., 2009). A decline in Arc and Egr1 expression levels was correlated with the occurrence of cognitive impairment during aging (Blalock et al., 2003) and in transgenic mice carrying genes responsible for AD (Dickey et al., 2003, 2004). Aβ oligomers can inhibit BDNF-induced Arc expression in culture cortical neurons (Wang et al., 2006; Echeverria et al., 2007), an effect modulated NMDA activity (Shankar et al., 2007). Because these genes are well-known to interact together, their low expression levels together with Aβ accumulation may explain the progressive cognitive decline of APP_Swe_/PS1 mice.

This accumulation of soluble Aβ could result of an enhanced production, a reduced degradation and/or a disrupted clearance. A major phenomenon in AD is the inability of mononuclear phagocytes to clear Aβ. Indeed, transplantation of competent CCR2 hematopoietic stem cells is able to counteract amyloid pathology and to restore mnesic capacity in 6-month-old APP_Swe_/PS1 and APP_Swe_/PS1/CCR2^−/−^ mice (Naert and Rivest, 2012). In addition, CCR2 gene delivery in cells of the bone marrow restored the memory and learning capacities of 6-month-old APP_Swe_/PS1 mice (Naert and Rivest, 2012), suggesting strongly that there is dysfunctional CCR2 system in the bone marrow of APP_Swe_/PS1 mice. For the first time we demonstrated that 6-month-old APP_Swe_/PS1 mice exhibited in the bloodstream a significant reduction of monocyte frequency, resulting of monocytopenia in the CX_3_CR1^low^Ly6-C^high^Gr1^+^CCR2^+^ subset. As we have previously observed at the age of 3–4 months (Michaud et al., 2011; Naert and Rivest, 2012), 5-week-old WT and APP_Swe_/PS1 mice exhibited similar monocyte frequency for both subsets. In addition, the CX_3_CR1^high^Ly6-C^low^Gr1^−^CCR2^−^ subset has similar frequency in 6-month-old APP_Swe_/PS1 mice than in 6-month-old WT mice and this at all ages studied (Michaud et al., 2011; Naert and Rivest, 2012). As we only observed an effect in one of the two-monocyte subsets and not in the whole monocyte population, a proper effect of the presence of APP transgene controlled by the prion promoter seems to be excluded, suggesting strongly a preferential effect of Aβ on inflammatory monocytes.

Therefore, this reduced level of inflammatory monocytes in bloodstream seems to be CCR2-dependent and could be explained by a reduced egress of monocyte from the bone marrow to blood circulation. Indeed, one major function of CCR2 is to permit the migration of CCR2^+^ monocytes from bone marrow to the blood (Serbina and Pamer, 2006; Engel et al., 2008). In addition CCR2 mediates hematopoietic stem and progenitor cell trafficking to sites of inflammation and monocyte infiltration into brain (Izikson et al., 2000; Babcock et al., 2003; Si et al., 2010). As the ratio of Gr1^+^/Gr1^−^ monocytes is significantly decreased in a context of AD, less CX_3_CR1^low^Ly6-C^high^Gr1^+^CCR2^+^ monocytes are available to clear Aβ in the bloodstream but also to be recruited into brain to remove brain Aβ. This expansion of the non-classical CD14^+^CD16^+^ subtype—counterpart of mouse CX_3_CR1^high^Ly6-C^low^Gr1^−^CCR2^−^—has been already observed in aged healthy adult (Seidler et al., 2010). In addition their phenotype changes dependent on age, resulting in lower expression of activation markers and chemokine receptors (Seidler et al., 2010). The CD14^++^CD16^−^ monocytes display some variation in CCR2 expression, with highest levels in healthy adults at the age between 30 and 50 years and a significant decreased expression in adults older than 50 years (Seidler et al., 2010). Although alterations in peripheral immune cells are also reported in patients with AD, examination of peripheral leukocytes—principally lymphocytes and monocytes—by different groups revealed conflicting results and currently there is no general consensus on the modifications of leukocytes in AD patients. By contrast, a recent analysis of peripheral blood mononuclear cell (PBMC) in a large older human population revealed increased of CCR2 levels associated with lower Mini Mental State Examination (MMSE) scores (Harries et al., 2012). These data corroborates the increased CCR2 expression in PBMC of AD patients (Reale et al., 2008; Pellicano et al., 2010). It is important to mention that CCR2 expression levels were only determined in lymphocytes (Reale et al., 2008; Pellicano et al., 2010). CCR2 expression is globally upregulated in CD4^+^ lymphocytes and down regulated in CD8^+^ lymphocytes (Reale et al., 2008). Unfortunately these studies have not investigated the CCR2 status in monocytes of AD patients neither that they did perform the distribution of each monocyte subset. The global increase of CCR2 expression levels in PBMC could then reflect CCR2 upregulation in lymphocyte, considering the prevalence of lymphocytes in human PBMC. More work has to be done to better understand the role of CCR2 in monocytic cells in AD patients.

Here analysis of CCR2 levels in FACS studies did not reveal a different intensity in monocytes between 6-month-old WT and APP_Swe_/PS1 mice (data not shown), suggesting that their low blood frequencies may be caused by a decreased production or their retention in bone marrow as observed in CCR2-deficient mice (Serbina and Pamer, 2006). We found a decreased frequency of Ly6-C^high^ monocytes in the bone marrow of 6-month-old APP_Swe_/PS1 mice, suggesting a deficient production of Ly6-C^high^ monocytes. These data support the concept that alteration in the numbers, phenotype, and functionality of mononuclear cells occurred in AD. Indeed monocytes of AD patients exhibit poor *in vitro* differentiation, poor phagocytic properties for and undergo apoptosis after Aβ application (Fiala et al., 2005).

The development of blood monocytes is dependent on M-CSF (also known as Csf-1) (Auffray et al., 2009). In mice deficient in M-CSF and its receptor M-CSFR (CD115), monocyte frequency is drastically reduced in bloodstream (Ryan et al., 2001; Dai et al., 2002). M-CSF transgene expression rescues the differentiation of monocytes in M-CSF-deficient mice (Ryan et al., 2001). It is very interesting to note that a short systemic treatment with M-CSF was able to prevent and rescue cognitive impairments in APP_Swe_/PS1 mice and reestablish the circulating level of monocytes. The defect in mononuclear cells observed in APP_Swe_/PS1 mice is therefore not dependent on CD115, since M-CSF treatment increased monocyte frequency in 4-month-old APP_Swe_/PS1 mice in levels comparable to those of age-matched WT mice. M-CSF treatment failed to increase monocyte population in CCR2^−/−^ and APP_Swe_/PS1/CCR2^−/−^ mice, suggesting a CCR2-dependent mechanism of M-CSF. These data suggest that expression of CCR2 is low in the bone marrow of APP_Swe_/PS1 mice or Ly6-C^high^ monocyte differentiation is impaired, and this may explain the low frequency of inflammatory monocytes, but these cells still have the ability to respond in presence of hematopoietic cytokines. These data obtained after 4-day M-CSF treatment confirm our previous results observed after a long M-CSF treatment in APP_Swe_/PS1 mice (Boissonneault et al., 2009). Indeed a long M-CSF treatment prevented and restored cognitive deficits, but M-CSF also increased the number of microglia and decreased the number of Aβ deposits (Boissonneault et al., 2009), certainly by enhancing Aβ phagocytosis by microglia, a known effect of M-CSF (Mitrasinovic and Murphy, 2003; Mitrasinovic et al., 2003; Majumdar et al., 2007). Besides, other hematopoietic cytokines, such as G-CSF or GM-CSF, had been shown to have beneficial effects in AD mouse model (Tsai et al., 2007; Volmar et al., 2008; Sanchez-Ramos et al., 2009; Boyd et al., 2010; Jiang et al., 2010; Li et al., 2011). More interestingly, G-CSF decreased both brain Aβ deposition and soluble Aβ accumulation by activating directly microglia and by enhancing the recruitment of BMDM and also restored synaptophysin expression in APP mice (Sanchez-Ramos et al., 2009). Despite a general beneficial therapeutic effect of most of hematopoietic cytokines, M-CSF seems to be a preferential target in AD as M-CSF acts specifically on bone marrow to produce the monocytes, the microglial precursor cells. Although APP_Swe_/PS1 mice exhibited with aging a significant monocytopenia, their bone marrow cells are still able to respond properly to M-CSF treatment, suggesting a functional CSF1-R. Monocyte development and differentiation (Auffray et al., 2009) and Ly6-C^high^ monocyte extravasation (Tagliani et al., 2011) are dependent on M-CSF.

A defect in the monocytopoiesis was found in a mouse model of AD, resulting in a decrease of CX_3_CR1^low^Ly6-C^high^Gr1^+^CCR2^+^ subset of monocytes. This phenomenon may be a key mechansism for the accumulation of Aβ, synaptic disruption, deficit in neurotrophic factors, and decline of memory and learning functions. Stimulating the production of CCR2^+^ monocytes with M-CSF should be seriously considered for clinical trials in early diagnosed AD patients.

### Conflict of interest statement

The authors declare that the research was conducted in the absence of any commercial or financial relationships that could be construed as a potential conflict of interest.
